# Post-Partum Management of the Parturient State

**Published:** 1882-04

**Authors:** William M. Polk

**Affiliations:** Professor of Obstetrics in the University of the City of New York


### THE POST PARTUM MANAGEMENT OF THE PARTURIENT STATE.

Abstract of a Lecture by WILLIAM M. POLK, M.D., Professor of Obstetrics in the University of the City of New York.

Immediately after labor the condition of the blood, of course, differs little from what it was before that occurrence; but within a short time, within a few hours afterward, it contains a larger proportion of excrementitious material. The large amount of material which before went to the nourishment of the child is no longer used for this purpose, but is turned directly upon the mother's circulation, and as it contains a large amount of fibrin and excrementitious material, it must be gotten rid of by the excretory organs, else it will act as a poison. The state of the nervous system will differ in different patients acccording to their individual nervous organization. There is always a certain amount of shock consequent upon delivery; the greater the amount of suffering the greater the amount of shock. The shock of labor differs in no respect from the shock which you are familiar with in surgical procedures; of course, however, that of labor, unless that after instrumental delivery, is rarely so great as that after severe surgical operations; but it is simply a difference in degree, not of kind. The shock which occurs after a simple labor, is usually not sufficient to cause any great amount of apprehension, but that which occurs after graver operations, as version or craniotomy, is sometimes sufficient to terminate fatally shortly after delivery. In fact, a number of cases have been recorded in which the sole cause of death was shock which, as already said, was identical with surgical shock. You are therefore to give the patient that attention which will enable her quickly to rally from the effect produced upon her nervous system. Extreme shock, when present, indicated by clamminess of the surface, etc., demands stimulation.

The temperature of the parturient woman should not normally stand higher, during the first two days and a half, than from 98° to 99° F. If it go above that point, say to 100.5° or 101°, and remain there, you may be sure that some septic element is at work, and act accordingly.

About the third day after delivery there is a tendency to a slight elevation of the temperature, because of the incoming of the milk. In some women there will be no elevation of the temperature at this time whatever; in others, there will be rather a decided febrile reaction. The temperature may rise to 100°, or even to 101°. This, if it be due simply to the incoming of the milk, will subside as soon as the milk begins to flow; if it continue a longer time it is rather an indication of some degree of sepsis also.

A word or two with reference to the secretions in general. They are all increased immediately after delivery. You will find that the skin acts more freely than during labor; the flow of the urine is greater. Possibly the alimentary canal will show a disposition to act; but, although the excretions by way of this channel may be increased, as a rule, there is no outward evidence of this. The tendency after delivery is to constipation. The reason for this apparently is the habit of most women to constipation during pregnancy, and to the fact that as the child's head passes down through the pelvis it produces a certain amount of temporary paresis of the rectum by pressure. Because of this tendency to inactivity of the bowel after delivery, the old fashion was to administer a cathartic in the form of Epsom salts or castor oil, but the fastidiousness of the women of the present day rather leads them to favor the use of the enema, and since the trouble is chiefly due to inactivity of the rectum itself rather than to other parts of the alimentary canal, this method of relieving the constipation seems to be the more appropriate.

When you return to see the patient after the first twelve hours, make it your rule, as soon as you have felt the pulse, to inquire as to the condition of the bladder. You will find in not a few cases that there is a tendency to retention, even though labor may have been natural, and it is due to pressure of the child's head upon the bladder or the urethra during delivery, producing temporary paresis. If you find that the bladder has not acted, you must, of course, resort to some means to bring about its activity. There is a tendency of the bladder to empty itself at the same time that the rectum does; therefore, it is my rule, on visiting the patient twelve hours after confinement, and finding the bladder has not yet acted, to tell the nurse to administer an enema, using, if necessary, as much as three pints of water, or even four, to be injected slowly into the rectum, so that as much as possible may be retained. When that enema passes away it is found that in the large majority of cases the bladder also empties itself. In connection with the use of the enema, you can obtain beneficial results by applying hot cloths or a hot flax-seed poultice over the region of the bladder, which, by its relaxing effects, tends to correct any spasm which may exist and give rise to retention. If these remedies do not succeed, nothing will remain to be done but to introduce the catheter.

While speaking of the secretions and excretions of the parturient woman, it is proper to refer to the lochial discharge.

From the time that delivery is completed, there is for at least the first three or four days a bloody discharge—a sanious discharge, more properly speaking—which comes from the cavity of the uterus. After five or six days this discharge changes color, becoming greenish, and finally yellowish. It retains the appearance in general for the remaining three weeks of the parturient period; at the end of that time it usually ceases. If the bloody lochial discharge continue for a longer period than five or six days—say ten or fourteen days—the probabilities are that it is due to some abrasion of the cervix—a laceration, perhaps, from which there is an oozing of blood. Such a condition of things, it is likely, would call for treatment.

A part of this structure (the enlarged uterus), of course, as it undergoes fatty degeneration or involution, is carried off by the lymphatics and the blood-vessels into the general circulation, and is excreted through the kidneys, through the skin, and apparently through the alimentary canal. But a certain portion of it unquestionably passes out with the lochial discharge. The relation which the lochial discharge has to this process is perhaps not unlike that which the expectoration of sputa bears to the resolution of pneumonia.

It seems to me that no harm can come from the employment of the carbollized douche after every case of delivery, provided you take this precaution, to have each patient provided with her own glass tube or nozzle and syringe. There will then, it seems to me, be no danger of infection. Use then the douche in every case, commencing, say twenty-four hours after delivery. It is rarely necessary to begin sooner than this, because decomposition, except in rare cases, does not take place until the expiration of that poriod. But if, owing to the season of the year or some predisposition of the patient, you have reason to believe that decomposition will occur, or has occurred before that time, then do not hesitate to employ the douche sooner—even six or twelve hours after delivery. How to make intra-uterine injections I explained to you when speaking of septicæmia, and it is only in that state that you are to employ them. The average period of time that the patient is kept under observation is about nine days from delivery. Now, the wiser plan is this: to see the patient for six or nine days daily; see that her constitution is what it should be at that period; that she is in fair condition of strength, the appetite good, the functions being performed naturally. Say to her, however, I wish to see you about five or six weeks hence, in order to examine the condition of the genital passages, to find out whether there is remaining any laceration of the cervix, and whether the uterus is in a state of subinvolution. If you dismiss your patient six or nine days after delivery with the condition I speak of, possibly she will not come back into your hands, but pass into the hands of some specialist, who will call attention, perhaps, to a piece of bungling midwifery, which, of course, will not react very favorably upon your reputation. Now, it is unnecessary to say that the making of these cases of laceration may not be because of any special fault on your part, but that is not so much the point as is the fact that by seeing the woman after five or six weeks you may put her in a position to correct any difficulty that may be present.—*Med. Record.*

